# Euthanasia: A Controversial Entity Among Students of Karachi

**DOI:** 10.7759/cureus.1510

**Published:** 2017-07-24

**Authors:** Ameet Kumar, Syeda Naqvi, Pirthvi Raj Giyanwani, Fareeha Yousuf, Aaliya Masnoon, Kiran Bai, Deepak Kumar

**Affiliations:** 1 Department of Medicine, Macneal Hospital; 2 Jinnah Postgraduate Medical Centre, Jinnah Sindh Medical University (SMC); 3 Civil Hospital Karachi, Dow University of Health Sciences (DUHS), Karachi, Pakistan; 4 Civil Hospital Karachi, DOW Medical College; 5 Internal Medicine, Credit Valley Hospital, Mississauga, Ontario; 6 Medicine, Peoples University of Medical and health sciences for women; 7 Internal Medicine, Civil Hospital Sukkur

**Keywords:** ethics, research ethics, euthanasia, mercy killing

## Abstract

Background

A serene death may be achieved through skilled and compassionate care, as well as by the dying person's own sense of having lived a righteous life. The purpose of this study is to acquire information about students’ knowledge and understanding of euthanasia.

Materials and Methods

Four hundred and fifty-six students from four classes of two institutions with similar demographic characteristics were included in this cross-sectional study. A questionnaire adapted from a study of ‘Gruber, et al.’ was distributed among the respondents after obtaining a verbal informed consent. The questionnaire had two parts, first dealing with demographics of respondents, and in the second part students were given different situations and asked about their decision in that particular setting to understand their opinion about euthanasia.

Results

There were 31.7% medical students and 12.9% non-medical students in favor to provide complete medical information (p < 0.001) while 59.2% non-medical students thought that complete information should be given to a patient if any iatrogenic incident occurred. Same favored by 33.7% of medical students (p < 0.001). The majority of medical students (84.5%) felt that cardiopulmonary resuscitation (CPR) must always be provided (p < 0.001) and this was acceptable more among females (p = 0.001). Furthermore, medical students (57.6%) were more in favor of continuing maximum medical treatment including CPR than non-medical students (42.9%, p = 0.003). A total of 83% non-medical students and 46% medical students found euthanasia an acceptable practice.

Conclusion

Results show a significant difference in perception of medical and non-medical students regarding euthanasia. Non-medical students are more in favor of euthanasia than medical students. Also, it is observed that males seem to be more inclined towards euthanasia while females are more in favor to provide maximum medical treatment.

## Introduction

The passage from life to death should be serene and dignified, not an agonizing ordeal. A serene death may be achieved through skilled and compassionate care, as well as by the dying person's own sense of having lived a righteous life. There were circumstances, however, in which hastening the end of a life seemed the only apparent way to relieve suffering. Several countries or states have legislation permitting or decriminalizing euthanasia; these include Belgium, Finland, New Zealand, Netherlands, Norway, Sweden, Switzerland, Thailand and the United States [1-3].

Numerous studies have been done to understand the views of doctors, nurses and students regarding euthanasia. One study found that the factors associated with the wish to hasten death considered physical symptoms, psychological suffering, perceiving themselves as a burden to others, higher levels of demoralization, less confidence in symptom support, fewer social supports, less satisfaction with experiences and fewer religious beliefs [4]. A study on students in Germany showed that majority of the students wrongly assumed that physician-assisted suicide is a punishable offense. However, a narrow majority considered physician-assisted suicide ethically acceptable compared to euthanasia, more than twice as many participants considered physician-assisted suicide acceptable [5].

 A study conducted at the Chinese University of Hong Kong revealed that more non-medical students were in favor of administering the lethal dose of drugs to patients who will not be able to recover the good quality of life [6]. A study in Pakistan and India showed that the knowledge of doctors regarding euthanasia was insufficient and most of the opinions were controversial. Most doctors against euthanasia are adherent strictly to the cultural and religious beliefs; however, a research signifies religion has no influence on the practice [7-9]. Researchers from Aga Khan University found that there was ambiguity among physicians and nurses about this issue, but still, withdrawal of the life support was practiced by 83.2% doctors in ICU setting [10].

However, only a few studies have been conducted in Pakistan to know the view of Pakistani medical and non-medical undergraduates. The purpose of our study is to get views of students about euthanasia and to compare the views of medical and non-medical students [7,10].

## Materials and methods

This cross-sectional survey was conducted from January 2016 to July 2016 in two big institutions of Karachi. One was a medical university, Dow Medical College and other was a non-medical university, Wafaki University. Four hundred and fifty-six students, 147 non-medical students and 309 medical students took part in this study. A questionnaire was taken from a study conducted by Gruber, et al. on "Changes in medical students’ attitudes towards end-of-life decisions across different years of medical training" [6].

On the predetermined days, we approached these institutes twice a week and enrolled all willing students in the study who willingly enrolled in the study. Four classes of the students having similar demographic characteristics were selected and their details are provided in Table 1.

**Table 1:** Demographic characteristics of the respondents.

Students	Non-medical students	Medical students
No. of respondents (percentage)	147 (32.2)	309 (67.8)
Age (years) Mean ± SD	21.87 ± 2.087	20.45 ± 1.818
Female's number (%)	58 (39.5)	223 (72.2)
Male's number (%)	88 (59.9)	68 (22)

The questionnaire was distributed in the paper format after taking verbal consent in the class and the researchers gave a 10-minute pre-planned same presentation in each class explaining the definition of euthanasia, ICU, cardiopulmonary resuscitation (CPR) and also addressed the queries of the participants. The questionnaire had two sections: the first section dealt with demographics of respondents, and in the second section students were given different situations and asked about their decision in that particular setting to understand their opinion about euthanasia.

Data analysis was performed using SPSS 16 software package (IBM, Armonk, NY). Frequencies for each question were calculated in regard to positive responses. p-values were also computed by using chi-square for all questions. p-value <0.005 was considered significant.

## Results

Most of the students, both medical and non-medical, considered that patients should be admitted to ICU even if they have limited chance of survival (p = 0.587). In the case of patients where survival is not more than a few weeks, they should be admitted to ICU as per most of the medical students (69.6%) in comparison to non-medical students (53.7%, p < 0.001).

**Figure 1:**
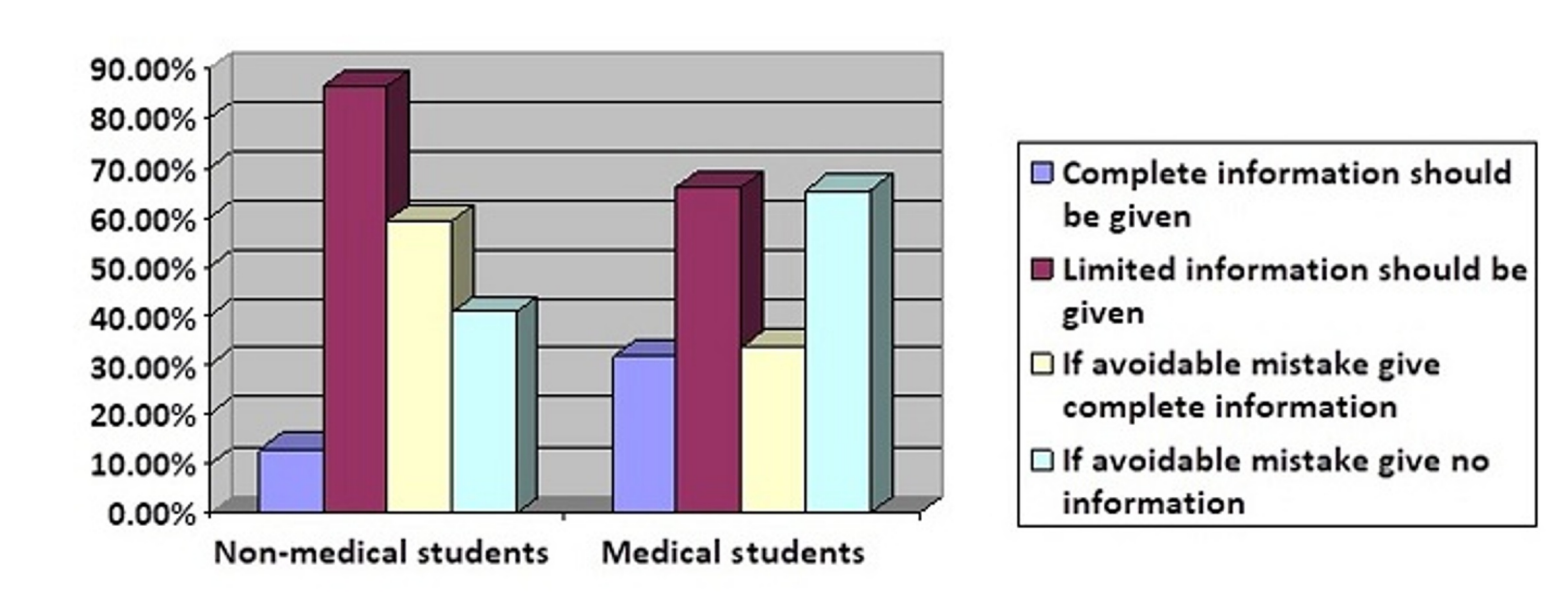
Information regarding avoidable mistake. Survey about amount of information to be given to patients and if any kind of avoidable mistake occurs then one should inform the patient or hide it.

In comparison to non-medical students, most of the medical students considered that complete medical knowledge should be given to the patient and family members (p < 0.001). In a case of an iatrogenic incident, most of the medical students and a few non-medical students think that patient should be informed in detail about the mistake (p < 0.001) as illustrated in Figure 1.

**Figure 2:**
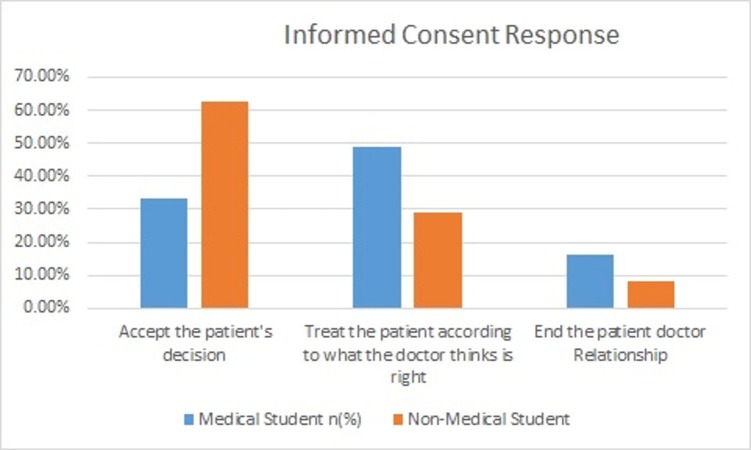
Doctor's response if the patient denies the necessary but life threatening surgery. In case of life threatening but necessary surgery, informed consent is obtained from the patient. Decision about the next step if the patient denies the procedure.

Most non-medical students believed that if a sufficiently capable person, the one who could understand his/her condition and could also decide on the basis of facts provided to him, refuses surgery that is necessary from the doctor's opinion (whether life-saving or not) the doctor should try to convince but the decision should be followed as per the patient’s choice (p =< 0.001). Non-medical students believed that one should try to accept the patient's decision for surgery if it is necessary but not life-saving. Medical students voted that one should treat the patient according to the doctor's decision. Results are shown in Figure 2.

**Figure 3:**
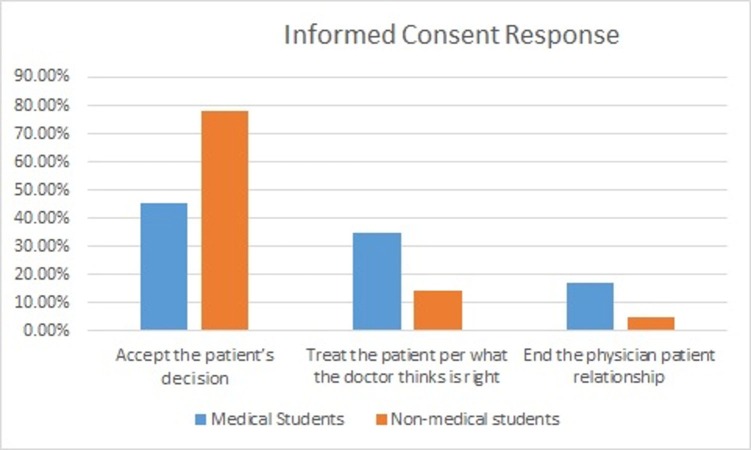
Doctor's response if the patient denies necessary but not life threatening surgery. In case of a necessary and relatively safe procedure, if the patient denies the surgery what should be the next step in view of medical and non-medical students?

In case, while obtaining informed consent of surgery which is necessary and life-saving then we had these options, i.e., treat the patient as per the doctor’s decision, accept the patient's decision or end the physician-patient relationship. So it was interesting that medical students think that the physician should end the relationship with the patient if he is denying a necessary and non-life threatening procedure. Responses of medical and non-medical students are shown in Figure 3.

**Figure 4:**
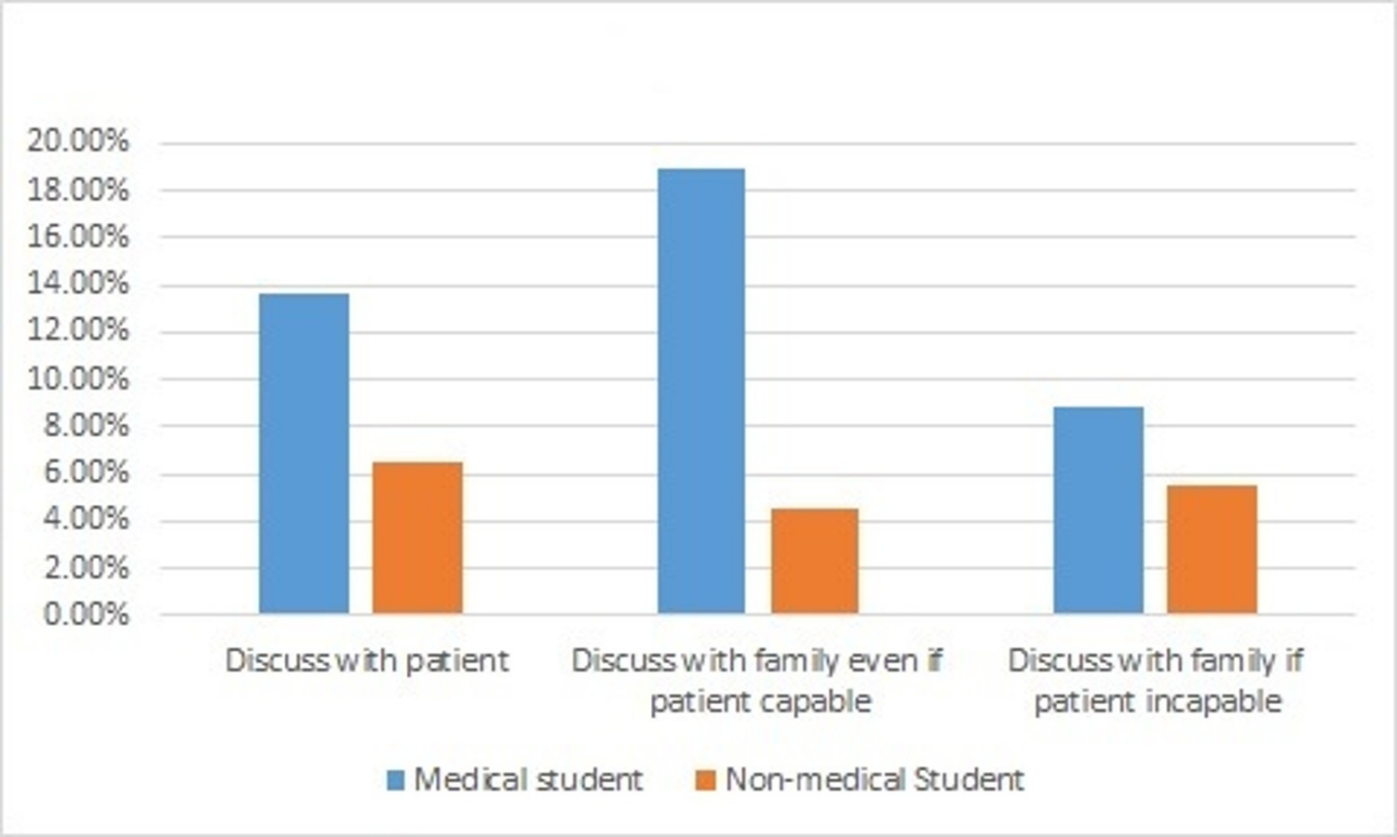
Decision regarding withholding cardiopulmonary resuscitation. About withholding cardiopulmonary resuscitation (CPR), who should be the primary person to take a decision?

Two-thirds of medical students (84.5%) and many of the non-medical students (70.7%) felt that CPR should always be provided (p =< 0.001). In conscious and mentally capable person, both medical and non-medical students thought to discuss with patients before deciding to withhold CPR (p = 0.618) but more of medical students felt that decision to withhold CPR should be discussed with families of conscious patients (p = 0.004). In incapable or unconscious patient, both medical and non-medical students thought that decision to withhold CPR should be discussed with families of patients (p = 0.095) as depicted in Figure 4.

**Figure 5:**
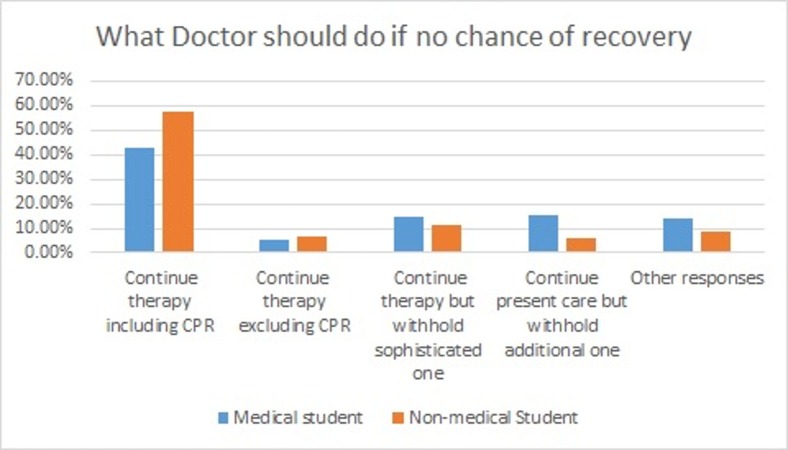
How to plan if no chance of recovery. In case of end-stage patient, what should be the final decision?

Almost half of the medical and non-medical students think that the treatment should not be withheld even if there is no chance of recovering (p = 0.009) but no difference was found in the attitudes of medical and non-medical students regarding discontinuation of therapy (p = 0.435) as shown in Figure 5.

## Discussion

In this study, we found a significant difference in the attitude of medical and non-medical students towards euthanasia. In regards to information delivery to patients and their family, most of the participants, both medical and non-medical, are generally in favor of providing knowledge depending on the type of patient and disease. This data is in concordance with a study conducted in Chinese Intensive Care medicine in Hong Kong [11-13].

When a competent patient refuses life-saving surgical interventions then a majority of the medical students were in favor of convincing the patient. While non-medical students think that patients should be treated as per doctor’s suggestion. This is because it is unethical in medicine to treat the patient against his/her will and most decisions are taken after having a consent and if the patient is not willing then doctors prefer to do counseling rather treating patients against their will.

The majority of medical students believe that CPR must be provided as compared to non-medical students, may be because non-medical students have less idea about CPR. Most of the students consider it as appropriate to withhold but inappropriate to discontinue treatment in patients in whom there is no chance of recovery.

In Islamic orientation paradigm, the strict opposition in the Holy Book (Qur'an) about suicide due to intolerable pain formed a strong opinion among Muslims that neither repentance nor the suffering of the person can remove the sin of suicide or mercy 'killing', even if these acts are committed with the purpose of relieving suffering and pain. Similar studies conducted in Turkey, Saudi Arabia, and other Muslim countries condemn euthanasia [14-18]. Although most of the participants of this study were Muslim, contrary to this a surprisingly large number of students found euthanasia acceptable and the majority of them are non-medical students (83%). Also in other religions like Christianity, there is an intense debate on euthanasia [19].

Research also showed that with increasing years of training physician-assisted suicide becomes less acceptable for students because medicine is based on the cure that is doctors prefer to save the life of patient until the end and this attitude of senior medical students is similar to qualified physicians who are strictly against such practice [20]. A similar study has been conducted in Australia and it showed a huge acceptance to euthanasia over time [21].

Most of the students chose to involve doctors, patients and their families in end-of-life decisions, and a very few chose nurses. This finding correlates with other research which found that in medical students’ opinion, patients should be told truth especially in the setting of the end-of-life decision [6] and this is in contrast to Cardoso, et al. who report that most of the times only medical team should be involved [22]. But according to most of the non-medical students, doctors should make decisions regarding end-of-life even if the patient is conscious and mentally capable.

This study has some limitations, for example, the questionnaire was designed in English, which was not the first language of non-medical students but the questionnaire was translated into their language. As this study is conducted in two big institutions, so these results can be assumed to be applied to all institutes of Pakistan but it is highly recommended to carry out such studies in order to determine widespread attitude on euthanasia.

## Conclusions

Considering euthanasia, there are a number of differences in the attitudes of medical and non-medical students. Studies, like this, are helpful in understanding different ethical perspectives and to design an ethical curriculum for medical professionals. This study shows that a sustainable impact on the end-of-life decisions can be expected if training is done compulsory in palliative medicine. This field should be a recognized one in those institutes without palliative department and extended in those institutes where it is a part of their curriculum.
